# Autoinflammatory diseases and the kidney

**DOI:** 10.1007/s12026-023-09375-3

**Published:** 2023-03-29

**Authors:** Mohamed Tharwat Hegazy, Ahmed Fayed, Rossana Nuzzolese, Jurgen Sota, Gaafar Ragab

**Affiliations:** 1 Rheumatology and Clinical Immunology Unit, Internal Medicine Department, Cairo University, Cairo, Egypt; 2 School of Medicine, Newgiza University (NGU), Giza, Egypt; 3 Nephrology Unit, Internal Medicine Department, Cairo University, Cairo, Egypt; 4 Research Center of Systemic Autoinflammatory Diseases and Behçet’s Disease Clinic, Department of Medical Sciences, Surgery and Neurosciences, University of Siena, Siena, Italy

**Keywords:** Autoinflammatory diseases (AIDs), Kidney involvement, Amyloidosis, Inflammasome, Nephropathy, Drug-induced nephrotoxicity, Interleukin-1 inhibitors

## Abstract

The kidney represents an important target of systemic inflammation. Its involvement in monogenic and multifactorial autoinflammatory diseases (AIDs) vary from peculiar and relatively frequent manifestations to some rare but severe features that may end up requiring transplantation. The pathogenetic background is also very heterogeneous ranging from amyloidosis to non-amyloid related damage rooted in inflammasome activation. Kidney involvement in monogenic and polygenic AIDs may present as renal amyloidosis, IgA nephropathy, and more rarely as various forms of glomerulonephritis (GN), namely segmental glomerulosclerosis, collapsing glomerulopathy, fibrillar, or membranoproliferative GN. Vascular disorders such as thrombosis or renal aneurysms and pseudoaneurysms may be encountered in patients with Behcet’s disease. Patients with AIDs should be routinely assessed for renal involvement. Screening with urinalysis, serum creatinine, 24-h urinary protein, microhematuria, and imaging studies should be carried out for early diagnosis. Awareness of drug-induced nephrotoxicity, drug-drug interactions as well as addressing the issue of proper renal adjustment of drug doses deserve a special mention and should always be considered when dealing with patients affected by AIDs. Finally, we will explore the role of IL-1 inhibitors in AIDs patients with renal involvement. Targeting IL-1 may indeed have the potential to successfully manage kidney disease and improve long-term prognosis of AIDs patients.

## Introduction

The concept of autoinflammation has arisen from the recognition of monogenic disorders with seemingly unprovoked inflammation without the high-titer autoantibodies or antigen-specific T cells usually detected in classic autoimmunity. While the firstly described diseases, also considered as inflammasomopathies, recognize interleukin-1 (IL-1) overproduction as their pathogenetic hallmark, recent evidence have expanded our knowledge by discovering other mechanisms involved in innate immune pathways including nuclear factor kappa B activation and type 1 interferon production [1, 2]. Autoinflammatory diseases (AIDs) may be divided into monogenic and polygenic autoinflammatory syndromes depending on their genetic background. Regarding the disease onset, in most cases the first manifestations emerge during childhood. However, adult onset is being increasingly recognized. From a clinical standpoint, although recurrent high-grade fever represents a common ground for most AIDs, their clinical presentation may be extremely heterogeneous, and virtually all organs and tissues may be potential targets of inflammation. Rash, serositis, arthritis, meningitis, uveitis, lymphadenopathy, and splenomegaly are frequently encountered [3, 4]. Most of these systemic features are the result of an increased IL-1β production. In this context, the kidney represents an important target of systemic inflammation. On one hand, amyloid deposition leading to organ failure due to physical replacement of parenchymal tissue by amyloid deposits is responsible for damage accrual. On the other hand, the role of nucleotide-binding oligomerization domain-like receptor family, pyrin domain-containing 3 (NLRP3) inflammasome in non-amyloid related damage appears to be more than marginal. Many damage-associated molecular patterns including reactive oxygen species, extracellular ATP, uric acid, nucleic acids, and extracellular matrix components released during renal injury are capable of activating the NLRP3 inflammasome with elevated levels of IL-1 and/or IL-18. This network ultimately escalates the inflammatory response and the subsequent development of fibrosis [5]. Kidney involvement that is relatively characteristic and frequent in some AIDs is uncommon and very heterogeneous in others.

In this review, we will unveil some pathogenetic insights and discuss renal involvement in the main monogenic and multifactorial AIDs. Finally, we will pay special attention to the potential attractiveness of IL-1 inhibition in chronic kidney disease.

## Pathogenetic insights

### Amyloid-related renal damage

With a continuous accumulation of serum amyloid A (SAA) protein, AA amyloidosis emerges in 5% of patients. Patients with a long track record of chronic inflammation (median 17 years), a significant acute phase SAA response, and homozygosity for the SAA1 genotype are at increased risk. Allelic heterogeneity at the SAA1 locus, which results in diverse genotypes, plays a role in either favoring or preventing amyloid formation [6, 7]. Genotyping for SAA1 may indeed provide valuable information for the management of patients with poorly controlled hereditary AIDs, allowing treatment decisions to be guided and SAA suppression to be targeted based on risk.

It may present either acutely or insidiously. Early signs of AA amyloidosis include low-grade proteinuria that normally develops over time if the inflammatory stimulus persists or recurs, eventually leading to nephrotic syndrome and renal failure. Advanced renal impairment at diagnosis, as well as continuous elevation of SAA during follow-up, are strong predictors of progression to end stage renal disease (ESRD) and death [7]**.**

**Fig. 1:**
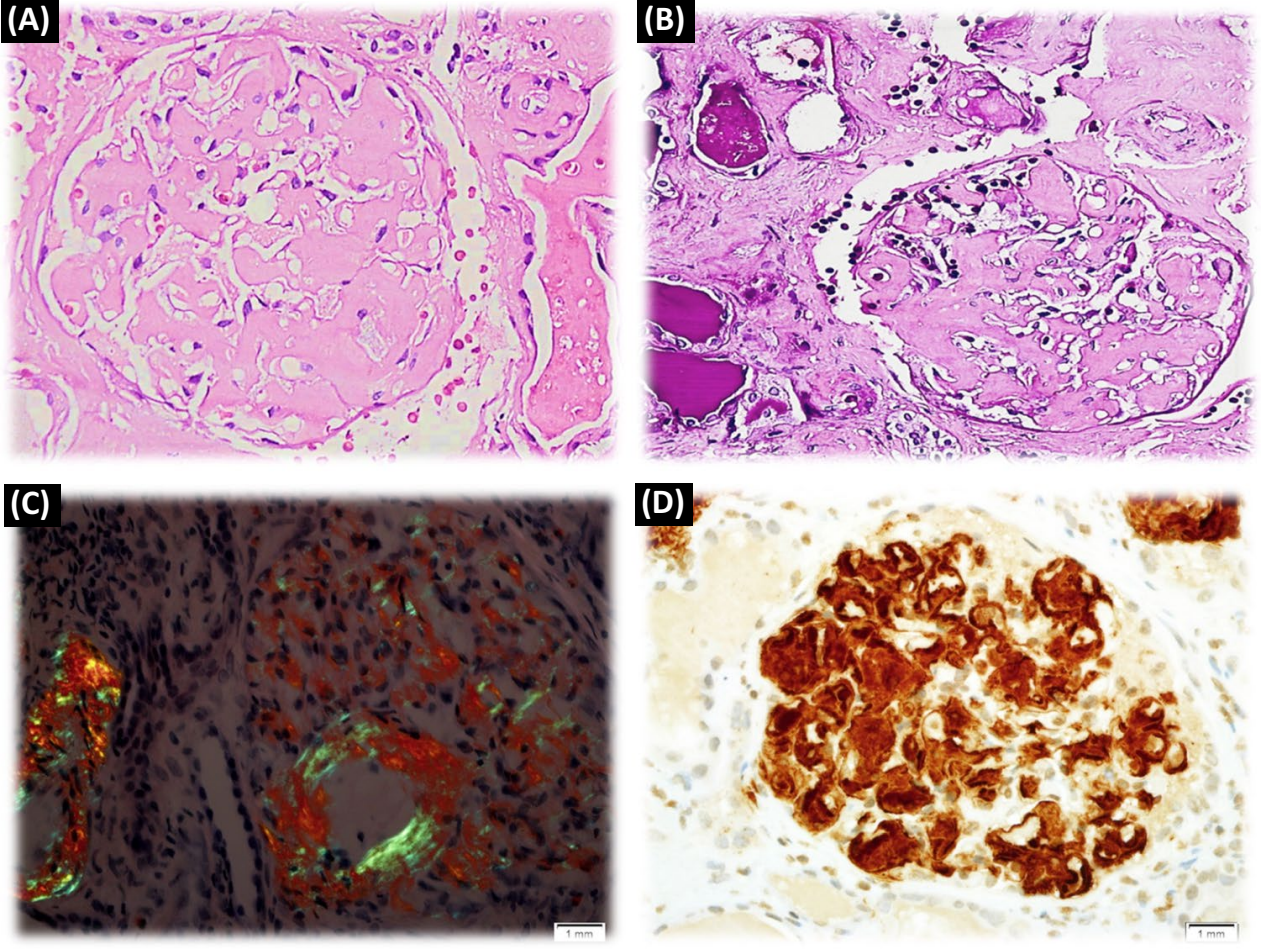
(A: H&E – ×400): 55 years male patient with FMF. A glomerulus shows abundant mesangial as well as capillary wall deposits of amorphous eosinophilic material. (B: PAS-×400): The deposited material is PAS negative. (C: Congo red-×400): Congo red stain reveals organophilic glomerular deposits; with polarized light an apple green birefringence is seen. (D: Immunoperoxidase, AA protein-x400): The tufts are strongly positive for amyloid associated protein (Courtesy of Dr. Wael M. Hamza, Department of Pathology, Cairo University)

Congo red–positivity denotes amyloid deposition, and to confirm that it is of the AA type, immunohistochemical staining with antibodies to SAA is necessary for conjunction with evidence of amyloid deposits in a tissue biopsy (Fig. 1). This emphasizes the importance of histological evidence of amyloid accumulation in all AID patients who develop renal impairment. To reduce bleeding risks, non-invasive procedures such as an abdominal fat aspirate is preferred. A negative test however does not exclude AA amyloidosis and a rectal or labial salivary gland’s biopsy represents a practical alternative [8].

In patients with hereditary AIDs, AA amyloidosis prevention is required. Although there are no evidence-based guidelines for effective monitoring of AA risk, accurate surveillance can be accomplished by measuring inflammatory markers and microalbuminuria during symptom-free intervals. Colchicine can help patients with non-nephrotic proteinuria avoid developing nephrotic-range proteinuria, as well as reverse nephrotic syndrome and prevent eGFR deterioration. Second-line agents are represented by anti–IL-1 or anti-TNF-α biological drugs that may help unresponsive or multidrug intolerant patients [6].

### NLRP-3 and inflammasome in renal disease

Previous research has found a link between kidney disease and inflammasomes, which could be direct or indirect. NLRP3 inflammasome is a sensor of kidney injury, both of tubular and glomerular origin and its activation fuels downstream canonical signaling pathways with overproduction of IL-1β and IL-18. However, non-canonical pathways, particularly those involving TGF-beta may be involved. Indeed, NLRP3 appears to be involved in the transition of tubular epithelial cells into epithelial-mesenchymal cells that leads to tubular atrophy and progressive interstitial fibrosis in chronic kidney disease[9]. Circulating levels of both the proinflammatory cytokine IL-1 and particularly its receptor antagonist (IL-1Ra) are elevated in chronic kidney disease. IL-1Ra levels have been found to significantly increase since the earliest stage of renal failure [10, 11].

The inflammasome is activated in chronic kidney disease regardless of the etiology [5]. Preclinical evidence and animal models on IL-1 role in chronic kidney disease point out its attractiveness as a therapeutic target. Furthermore, in experimental mesangioproliferative nephritis, IL-1Ra inhibits mesangial and glomerular macrophage infiltration [12]. In experimental anti-glomerular basement membrane GN, IL-1 can directly up-regulate renal osteopontin expression. In contrast, IL-1Ra reduces osteopontin expression in glomeruli and tubules, decreases macrophage accumulation and progressive renal injury during both the induction phase and the established disease [13]. In murine anti-myeloperoxidase antibody-induced GN, neutrophil serine proteases-dependent IL-1ß generation was an important step in the development of necrotizing crescentic GN while the IL-1 receptor antagonism was protective [14]. IL-1ß synergizes with angiotensin II to promote NF-B activation and a proinflammatory reaction marked by increased MCP-1 and IL-6 production [15]. Hyperhomocysteinemia (hHcys)-induced glomerular injury, whether or not associated with hypertension, was established in vitro by the development and activation of the NLRP3 inflammasome complex in podocytes, resulting in podocyte injury and glomerular inflammation, which led to glomerulosclerosis. Several studies have suggested that hHcys is an important contributor to the development and progression of renal injury linked to local inflammation, oxidative stress, poor cell metabolism, and fibrogenesis; indeed, NLRP3 is activated in podocytes during hHcys. The generation of reactive oxygen species (ROS) generated in hHcys has been linked to NLRP3 activation in podocytes and, ultimately, glomerular damage [16, 17].

Podocytes are a key component of the glomerular filtration barrier, and podocyte injury affects proteinuria. So, studying inflammasome in podocytes can provide new target and a possible treatment of renal diseases [17].

### Immunoglobulin A nephropathy

**Fig. 2:**
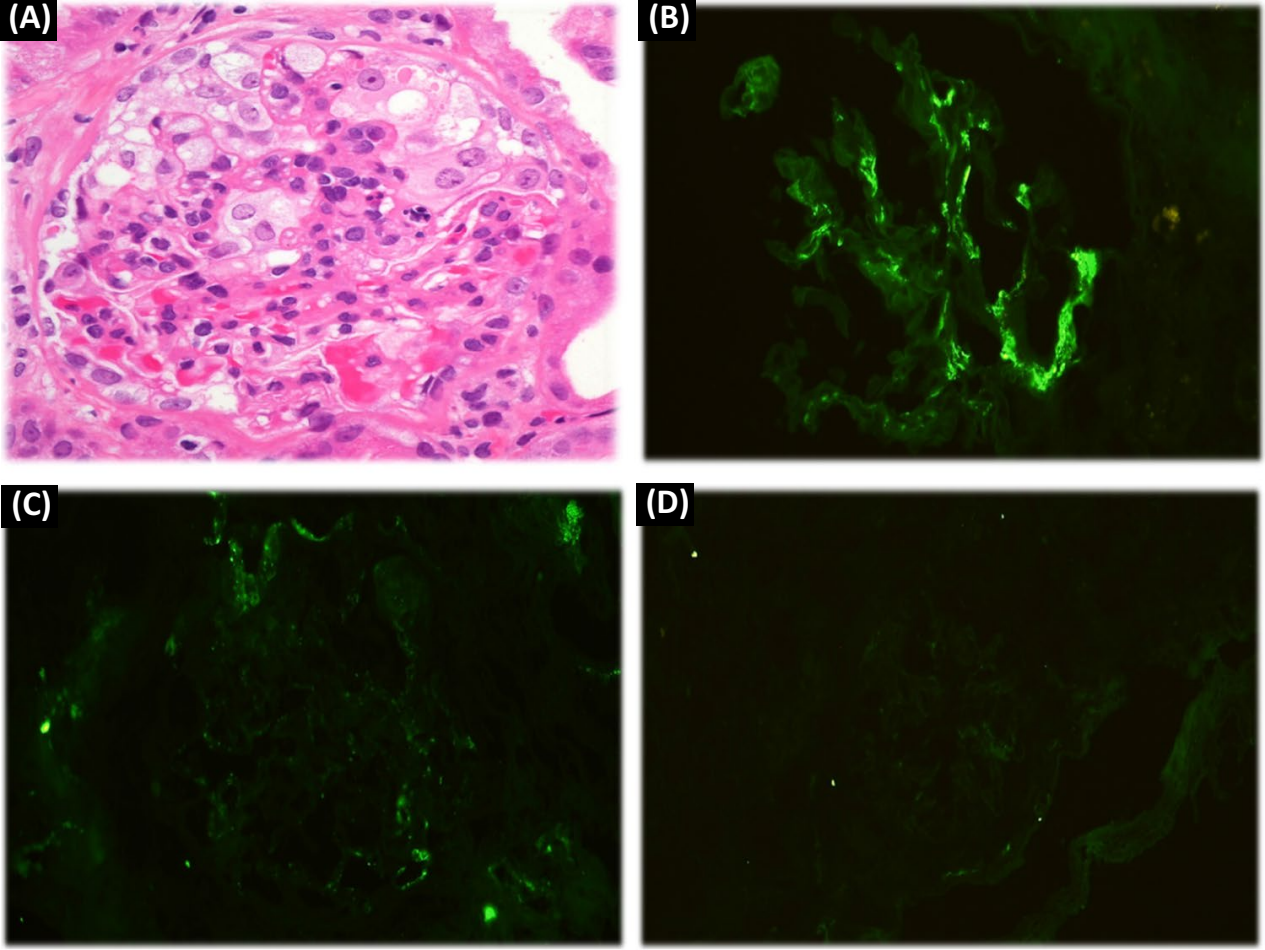
(A: H&E – ×400): 38 years male patient with FMF. A glomerulus shows mesangial hypercellularity and hypertrophied epithelial cells. Increased intraluminal leukocytes and congestion are also evident. (B: IF, IgA-×400): The immunofluorescence study reveals dominant mesangial deposits for IgA. (C &D: IF, C3 & IgG-×400): Subtle deposits of C3 (image C), while IgG was negative (image D). (Courtesy of Dr. Wael M. Hamza, Department of Pathology, Cairo University)

Immunoglobulin A nephropathy (IgAN) is the most frequent primary glomerulonephritis [18] (Fig. 2).

The main pathogenetic hypothesis highlights the abnormal glycosylation of IgA which alone is not sufficient to induce kidney injury. In fact, specific IgA and IgG autoantibodies recognize the underglycosylated IgA molecule that in turn determine the formation of immune complexes with local activation of the complement system. This will eventually translate into varying degrees of intrinsic cell proliferation, particularly in mesangial cells, kidney injury, fibrosis, and ESRD in up to 40% of patients [19]. Interestingly, an association between Henoch-Schonlein purpura (also known as IgA vasculitis) and FMF has been reported [20].

A) Role of NLRP3 inflammasome in IgA nephropathy

Several studies have recently suggested that the NLRP3 inflammasome may play a pathogenetic role in IgAN. In renal biopsies of individuals with IgAN, NLRP3 gene expression was shown to be higher than in control kidneys and to be associated with clinical outcomes [21]. IgA immune complexes can activate the inflammasome in macrophages via ROS and stimulate the production of IL-1β and caspase-1, suggesting that NLRP3 may play a role in IgAN. In addition, factors that modulate NLRP3 activation can alleviate IgA inflammation to some extent. However, more work remains to be done to understand the inflammasome's exact mechanism in IgAN, such as the link between IgA-related immune response and the inflammasome. There will be a need to develop viable therapeutic options [22]. Also, it is still unclear wether the inflammasome activation is directly responsible for more severe cases of IgAN, namely patients that present a bioptic milieu characterized by mesangial hypercellularity, segmental glomerulosclerosis and tubular atrophy/interstitial fibrosis. Interestingly, the location of IgA deposits in the glomerular capillary wall has been suggested to associate with a worse prognosis than mesangial deposits alone. Altogether, these data may advocate for a more aggressive immunosuppressive treatment. To this end, the role of IL-1 inhibitors is worth investigating [23].

B) The role of the complement system in IgA nephropathy

The complement system comprises the frontline of the innate immune system. Understanding its role in IgA nephropathy may offer a potential possibility for complement-targeting therapy of the disease.

Several complement proteins, mostly C3, less frequently C4 and rarely C1q were detected in IgA immune complexes in the mesangium sharing in renal injury [24].

## Renal involvement in the four canonical monogenic AIDs

### Familial Mediterranean fever (Figs. 1, 2, 3)

FMF is the leading cause of secondary amyloidosis and represents a specific type of AA amyloidosis. FMF is usually passed down as an autosomal recessive condition, and it mostly affects Arabs, Sephardic Jews, and Armenians. Genotype affects the phenotype regarding the clinical manifestations, severity of the disease, and response to colchicine [20].

**Fig. 3:**
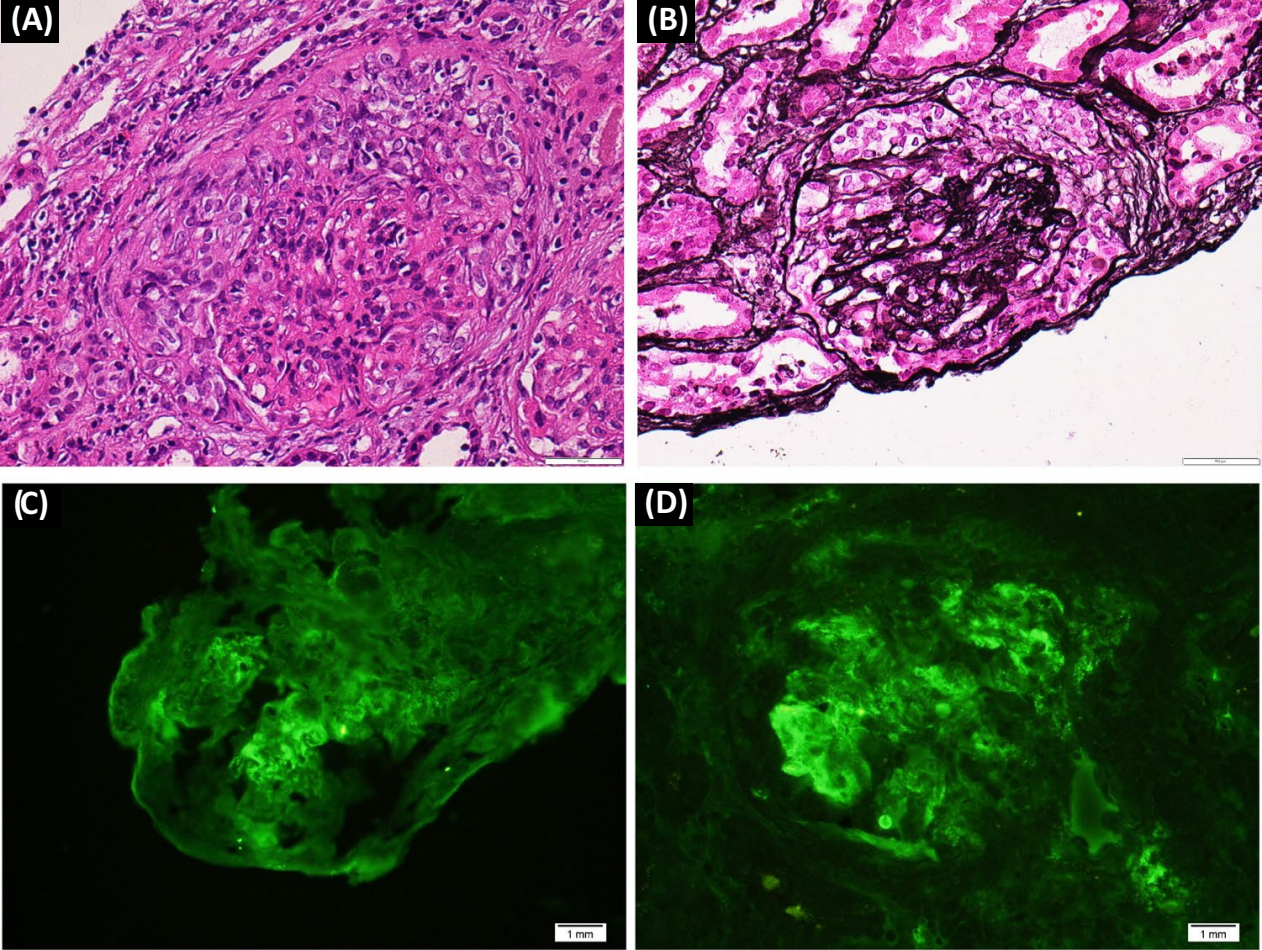
(A: H&E & B: JMS – ×400): 32 years female patient with FMF. Two glomeruli show compressed tufts by cellular crescents. (C & D: IF-×400): Immunofluorescence study reveals positive glomerular deposits for IgG (image C) and IgM (image D). (Courtesy of Dr. Wael M. Hamza, Department of Pathology, Cairo University)

There are three phenotypes in clinical practice. Phenotype 1: Brief episodes of febrile peritonitis, pleuritis, or synovitis that occur in childhood or adolescence and occur before renal symptoms. Phenotype 2: Renal symptoms precede other manifestations and maybe the only indication of the disease. Phenotype 3 is represented by patients carrying mutations in MEFV gene in the absence of typical clinical manifestations. Acute phase reactants, such as SAA, are dramatically elevated during the attacks. Amyloid deposits cause severe kidney affection marked by substantial glomerular pathology, which can lead to ESRD and early death [25].

Amyloidosis is the commonest cause of kidney involvement in FMF patients. Many types of glomerulonephritis (GN) have also been reported. IgA nephropathy, focal GN, and mesangioproliferative GN were the most prevalent non-amyloid glomerular lesions [26].

### TNF receptor-associated periodic syndrome (TRAPS)

In TRAPS, AA amyloidosis occurs in up to 25% of the affected patients, leading to renal amyloidosis [27]. Also, IgAN has been reported [28].

### Cryopyrin-associated periodic syndromes (CAPS)

It is accompanied by high levels of SAA and long-standing inflammation which may lead to renal amyloidosis [29].

### Mevalonate kinase deficiency

It is a less severe form of a metabolic disorder called mevalonate kinase deficiency (MKD). Renal involvement may result from AA-amyloidosis or glomerulonephritis [30].

## Other monogenic AIDs

Proline-serine-threonine phosphatase-interacting protein 1 (PSTPIP1)-associated myeloid-related proteinemia inflammatory (PAMI) syndrome represents the most severe form of PAID spectrum. Borgia P. et al presented a case of PAMI syndrome associated with nephrotic-range proteinuria and normal kidney function. Needle renal biopsy revealed focal segmental glomerulosclerosis while interstitial fibrosis was detected on a second biopsy performed due to persistence of proteinuria. They subsequently performed a literature review and found other five patients with the PAMI-associated PSTPIP1 mutations displaying renal affection with at least three different phenotypes: glomerular vasculitis in three patients, tubulointerstitial infiltration likely due to a persistent inflammatory state in one patient as well as glomerular calprotectin deposition in the last case. Of note, not a single case was found to have amyloid or immune complex deposition suggesting that other pathogenetic mechanisms apart from IL-1 overproduction may be implicated [31].

COPA syndrome is a rare disease with autoimmune and autoinflammatory traits caused by heterozygous loss-of-function mutations in the coatomer subunit alpha (*COPA*) gene and considered as a monogenic interferonopathy. Patients affected by COPA syndrome are at high risk of renal involvement with almost half of the patients exhibiting glomerular disease testified by proteinuria and/or impaired kidney function. Bioptic findings are very heterogeneous including crescentic disease, IgA nephropathy with necrotizing lesions and mesangial hypercellularity lacking immune deposits [32]. Lupus-like nephritis has also been encountered [33]. Despite the absence of characteristic and distinct pathological features, renal involvement can be severe [32].

Two cases of collapsing glomerulopathy have been reported in one patient with SAVI (STING-associated vasculopathy with onset in infancy) syndrome and in one patient with Aicardi Goutières syndrome, suggesting that type 1 interferon response may be a leading driver in this type of involvement [34, 35].

## Renal involvement in multifactorial AIDs

### Behçet’s disease

The main challenge when trying to describe several aspects of kidney involvement in Behçet’s disease (BD) is the lack of robust evidence and only a few limited number of randomized clinical trials comes as a natural consequence of the rarity of BD, the relatively uncommon renal manifestations, and the absence of standardized protocols to define kidney disease in BD in terms of diagnosis, severity, and long-term outcomes. Despite the rarity of reports on renal involvement, urine abnormalities are not uncommon. Shahram’s study on 4386 patients found urinary abnormalities in 10.8% of patients that were transient in most cases. Hematuria, proteinuria, and renal casts were detected in 222, 101 and 13 patients, respectively [36].

The clinical spectrum of renal BD is heterogeneous, ranging from asymptomatic hematuria and/or proteinuria to ESRD. AA Amyloidosis, GN, and vascular abnormalities were found to cause renal BD [37]. Zheng et al on the other hand reported a series of 16 patients describing chronic GN in six patients, renal tubular acidosis in one case, renal artery stenosis in eight and renal venous thrombosis in a single case [38]. The mean interval between the diagnosis of BD and the detection of kidney disease is roughly 8 to 11 years [37, 39].

Amyloidosis represented the most frequent type of specific renal disease in a series of 33 BD cases, with male gender being a risk factor for its development [37]. Kosemehmetoglu and colleagues in their monocentric experience retrospectively reviewed 220 cases of secondary type amyloidosis over a 26-year period and reported an accompanying BD in 10 of them. Interestingly, all patients were males, supporting gender as a risk factor for developing renal amyloidosis [39]. SAA1 α/α genotype may represent an additional risk factor for BD-related amyloidosis given its frequency in this complication [40]. The morphology of renal amyloidosis caused by BD presented diversities in terms of amyloid deposition in renal compartments. In fact, glomerular amyloid deposition may be of hilar, mesangiocapillary or mesangial nodular type. Three out of four cases presenting with heavy proteinuria exceeding 5 g/day showed the mesangiocapillary type glomerular amyloid. Another finding deserving further investigation is the histologic feature of extensive involvement of renal arterioles with amyloid which appears to be associated with progression to ESRD [39].

Regarding GN, many types have been reported in BD without the predominance of a specific glomerular lesion. Anecdotal associations with IgA nephropathy and fibrillar GN have also been reported [41, 42]. It is possible for BD patients with renal involvement to experience subtype conversion over time, as shown by two cases of GN progressing to grades II and III IgA nephropathy [38]. However, given the rarity of these conditions it is still unclear whether their coexistence had a pathogenetic background or was purely coincidental. Additionally, diagnostic challenges may be encountered in selected cases. In particular, when extra-renal features are lacking or are non-specific, the presence of intrarenal aneurysm require a detailed differential diagnosis with polyarteritis nodosa [43]. Despite the several types of GN, including rare associations, prognosis of patients with GN is generally favorable and ESRD has been reported to develop only in a few cases [37].

Thrombosis, most frequently presenting as deep and/or superficial vein thrombosis of the lower limbs, is the most vascular involvement and weighs heavily from a prognostic point of view. Renal vein thrombosis represented 4.3% of a Chinese cohort of 93 BD patients who experienced thrombosis [44]. Another Chinese case series reported 16 BD patients with renal involvement with only one experiencing renal thrombosis whereas 8 patients had renal artery stenosis [38]. Long-term subclinical vasculitic changes can lead to stenosis of renal artery with consequent compromise of renal blood flow which appears to be influenced by disease duration. This in turn can potentially activate the renin-angiotensin-aldosterone system ultimately leading to elevated blood pressure [45]. Renal aneurysms and pseudoaneurysms, on the other hand, may develop due to endarteritis obliterans of the vasa vasorum and intense inflammation of the media and adventitia resulting in distension of the vessel caused by destruction of the media and weakening of the arterial wall. Surgery should be considered for these lesions given the high risk of rupture that in case of pseudoaneurysms does not depend on the lesion’s size [46, 47].

Renal involvement may be more prevalent in BD than previously stated. Routine screening with urinalysis, serum creatinine, 24-hour urinary protein, microhematuria and imaging studies is mandatory to detect renal involvement in BD early. Interestingly, microalbuminuria has been suggested as an early marker of renal involvement and has been associated with neurologic manifestations [48].

Biopsy may sometimes be required to precisely classify specific renal lesions. However, the unwelcome possibility of a pathergy reaction should be kept in mind [49].

### Other multifactorial AIDs

The literature regarding renal involvement in Schnitzler syndrome is limited to small case reports. One patient was reported to have immune complex-mediated tubulointerstitial nephritis associated with light chain deposition. Other forms of renal involvement including membranous nephropathy and type I membranoproliferative GN have also been described [50, 51]. Despite being rare and not very characteristic of Schnitzler syndrome, renal disease can be severe, thus warranting systematic investigations for nephropathy markers in order to perform an early diagnosis which ultimately leads to an overall better prognosis [52]. Similarly, renal involvement in Still’s disease is rare. Anecdotal cases of membranous GN [53], collapsing glomerulopathy [54, 55], thrombotic thrombocytopenic purpura [56, 57], IgA nephropathy [58], necrotizing crescentic IgA GN [59] have been reported.

The risk of amyloidosis for uncontrolled active disease must always be taken into consideration [60, 61].

Regarding Periodic fever, aphthous stomatitis, pharyngitis, and adenitis syndrome (PFAPA), Sugimoto and coworkers presented a child with PFAPA and concurrent IgA nephropathy. Microscopic hematuria has been for many years associated with proteinuria. Examination of a renal biopsy specimen helped to diagnose IgA nephropathy. They treated their case with multi-drug combination and subsequent tonsillectomy resulting in satisfactory outcomes in both diseases, suggesting that their coexistence was not coincidental [62].

A recent published study reported cases with renal involvement as a unique manifestation of hemophagocytic syndrome [63].

## IL-1 targeting options in renal disease

Among the various disorders affecting the kidney, AA amyloidosis remains a serious challenge and specific therapy for its successful management has yet to be developed. Treatment largely depends on the underlying systemic disease. Early diagnosis and proper treatment of AIDs is imperative to avoid developing of AA amyloidosis [64]. For instance, patients with FMF-related amyloidosis were shown to experience the improvement or stabilization of kidney function and proteinuria following anakinra therapy [65, 66]. Yazilitas and colleagues also reported as a secondary outcome, an increase of glomerular filtration rate and proteinuria stabilization after 3 months in two out of three of their FMF cases affected by AA amyloidosis [67].

Preclinical evidence on IL-1 role in chronic kidney disease points out its attractiveness as a therapeutic target [12–15]. Experimental models find support by clinical evidence. Novak et al. performed the first randomized controlled trial of IL-1 inhibition in patients with chronic kidney disease not requiring chronic dialysis. They assessed endothelium dependent dilation and stiffness of large vessels as endpoints and found that inhibition of IL-1 with rilonacept improved endothelium-dependent dilation. As these are considered independent predictors of cardiovascular events, the authors hypothesized that as a consequence they may positively impact cardiovascular outcomes in patients with moderate-to-severe chronic nephropathy [68].

Interleukin-1 receptor antagonist anakinra improved glycated hemoglobin, beta-cell secretory function and reduced markers of systemic inflammation within 13 weeks in patients with type 2 diabetes mellitus [69]. This in turn has potential implications in diabetic renal disease since diabetes represents the most frequent cause of ESRD [70].

Anakinra has also been tested in hemodialysis patients. In a pilot trial by Hung and his group, 14 hemodialysis cases completed the trial in the anakinra arm and showed significant lowering of inflammatory biomarkers when compared with the placebo arm. Survival however was not assessed in their study as it requires long-term studies [71].

There is anecdotal use of anakinra in renal transplant patients, in the context of an underlying systemic disorder requiring IL-1 blockade. Anti-IL-1 therapy may also provide a protective effect in solid organ transplantation [72, 73]. Canakinumab was shown to be a safe and effective option in renal transplant recipients with FMF [74]. In their retrospective multicenter study, Loustau et al. noted that anakinra successfully suppressed gout flares and was well tolerated in patients with stage 4-5 chronic kidney disease and post-renal transplant patients [75]. This is highly interesting, given the hazards that may emerge with colchicine and CsA.

Targeting IL-1 may indeed have the potential to successfully manage kidney disease and improve long-term prognosis of AIDs patients. This approach is potentially expandable also in more frequent diseases which have an autoinflammatory mark in their pathogenetic background. More empowered studies designed to specifically address this issue are warranted.

## Conclusion

The kidney represents an important target in monogenic and multifactorial AIDs. Renal injury can be mediated by different pathogenetic mechanisms including amyloid-related damage as well as non-amyloid-related damage such as NLRP3 inflammasome activation. Nevertheless, the severity of renal impairment in some AIDs should direct physicians to perform a regular diagnostic work-up for nephropathy markers to early detect kidney involvement which ultimately leads to an overall better prognosis. Targeting IL-1 is a promising approach to manage kidney disease, hence improving the long-term prognosis of AIDs.

## Abbreviations

ACEI: Angiotensin-converting enzyme inhibitors
AIDs: autoinflammatory diseases
AIN: Acute interstitial nephritis
Anti-TNF-α: Anti tumour necrosis factor α
ARBs: Angiotensin receptor blockers
ATN: acute tubular necrosis
BD: Behçet’s disease
C3: Complement 3
CAPS: cryopyrin-associated periodic syndrome
CHF: congestive heart failure
CIN: chronic interstitial nephritis
COPA: coatomer subunit alpha
CrCl: creatinine clearance
CsA: Cyclosporine A
EMT: epithelial-mesenchymal transition
ESRD: End stage renal disease
FMF: Familial Mediterranean fever
GN: glomerulonephritis
hHcys: Hyperhomocysteinemia
IgA: immunoglobulin A
IgAN: Immunoglobulin A nephropathy
IL-1: Interleukin-1
IL-1Ra: Interleukin-1 receptor antagonist
JAK: janus kinase
MCP-1: Monocyte chemotactic protein-1
MKD: mevalonate kinase deficiency
NF-B activation: nuclear factor -enhancer of activated B cells
NLRP3: nucleotide-binding oligomerization domain-like receptor family, pyrin domain-containing 3
NSAIDs: Nonsteroidal anti-inflammatory drugs
PAMI: PSTPIP- associated myeloid-related inflammatory syndrome
PFAPA: Periodic fever, aphthous stomatitis, pharyngitis, and adenitis syndrome
PSTPIP1: Proline-serine-threonine phosphatase-interacting protein 1
ROS: reactive oxygen species
SAA: serum amyloid A
SAVI: STING-associated vasculopathy with onset in infancy
TRAPS: TNF receptor-associated periodic syndrome
